# Approaches to improve the quality of maternal and newborn health care: an overview of the evidence

**DOI:** 10.1186/1742-4755-11-S2-S1

**Published:** 2014-09-04

**Authors:** Anne Austin, Ana Langer, Rehana A Salam, Zohra S Lassi, Jai K Das, Zulfiqar A Bhutta

**Affiliations:** 1 Harvard School of Public Health, Boston, USA; 2 Division of Women & Child Health, Aga Khan University, Karachi, Pakistan; 3 Program for Global Pediatric Research, Hospital for Sick Children, Toronto

## Abstract

Despite progress in recent years, an estimated 273,500 women died as a result of maternal causes in 2010. The burden of these deaths is disproportionately bourne by women who reside in low income countries or belong to the poorest sectors of the population of middle or high income ones, and it is particularly acute in regions where access to and utilization of facility-based services for childbirth and newborn care is lowest. Evidence has shown that poor quality of facility-based care for these women and newborns is one of the major contributing factors for their elevated rates of morbidity and mortality. In addition, women who perceive the quality of facilty-based care to be poor,may choose to avoid facility-based deliveries, where life-saving interventions could be availble. In this context, understanding the underlying factors that impact the quality of facility-based services and assessing the effectiveness of interventions to improve the quality of care represent critical inputs for the improvement of maternal and newborn health. This series of five papers assesses and summarizes information from relevant systematic reviews on the impact of various approaches to improve the quality of care for women and newborns. The first paper outlines the conceptual framework that guided this study and the methodology used for selecting the reviews and for the analysis. The results are described in the following three papers, which highlight the evidence of interventions to improve the quality of maternal and newborn care at the community, district, and facility level. In the fifth and final paper of the series, the overall findings of the review are discussed, research gaps are identified, and recommendations proposed to impove the quality of maternal and newborn health care in resource-poor settings.

## Introduction

**Figure 1:**
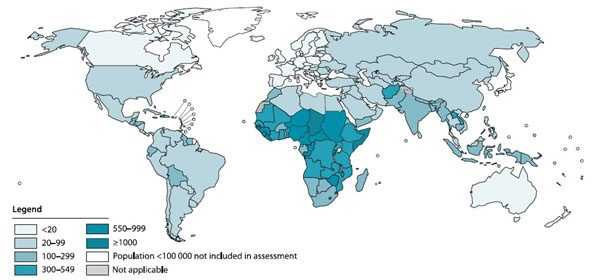
Map with countries by category according to their maternal mortality ratio (MMR, number of maternal deaths per 100 000 live births), 2010. Source: Trends in maternal mortality: 1990 to 2010 by WHO, UNICEF, UNFPA and The World Bank estimates [1]

Although there have been substantial declines in the annual number of maternal deaths since 1990, an estimated 273,500 women die every year as a result of maternal causes [1]. Sub-Saharan Africa (56%) and Southern Asia (29%) account for 85% of the global burden [2], while at the country level, two countries contribute a third of global maternal deaths: India at 19% (56 000) and Nigeria at 14% (40 000) (Figure 1). Among women who survive childbirth, approximately 10 million will suffer from complications related to pregnancy and childbirth [3,4]. Many of these conditions or deaths could be prevented through timely interventions that have proven to be effective and affordable [5-7].

Mortality in children under the age of five has also been reduced substantially and declined by over 47% since 1990. Unfortunately, neonatal mortality declines have lagged behind those of older children; the share of neonatal deaths among all under-five deaths has increased from about 36% in 1990 to about 44% in 2012 [8]. Neonatal outcomes are inextricably linked to maternal health and, therefore, to the quality of care a mother receives during labour, delivery and in the immediate postpartum period, the highest risk period for both mothers and babies. Maternal complications and maternal deaths significantly impact newborns’ ability to survive and thrive [9]. Neonatal deaths are concentrated in the same low and middle-income countries where maternal mortality is highest, facility utilization lowest, and the quality of available care poorest. For example, sub-Saharan Africa, where maternal mortality is the highest in the world (see above), also has the highest neonatal mortality rate (32 deaths per 1,000 live births in 2012) and is among the regions showing the least progress for over two decades [8].

There is evidence on the effectiveness of a number of interventions to prevent and manage all major causes of maternal morbidity and mortality, including good nutrition, access to contraception, skilled attendance at delivery, and emergency obstetric care [10]. Despite unquestionable evidence, the delivery and distribution of these services is uneven [10,11]. The safety and effectiveness of interventions to prevent the direct causes of newborn deaths, infections and asphyxia, are also well known. Regrettably, they are also unevenly delivered and not accessible to those who need them most [9]. Concern for these vulnerable populations should shape improved efforts to introduce and scale up the delivery of interventions that have been proven to be both safe and effective [12-14].

Even in contexts where efforts have focused on increasing access to institutional care, the expected improvements in maternal/newborn health have not materialized. In India and Ethiopia, two countries that account for one-fifth of global maternal deaths, large investments in infrastructure and provider training have not yet yielded the expected improvements in maternal and newborn health [2]. In India, an ambitious conditional cash transfer program, Janani Suraksha Yojana (JSY), has resulted in a significant increase in the number of institutional births, but it is unclear whether this has actually resulted in improved maternal health outcomes [15].

Ethiopia presents a different scenario: historically, the Health Sector Development programs have focused on improving the facility infrastructure, training health care providers and promoting referrals to health facilities for birth. Despite these efforts, only 10% of Ethiopian women have facility-based births [16]. In this context, maternal mortality has remained persistently high: the 2011 Ethiopian Demographic and Health Survey (EDHS) estimated a maternal mortality ratio (MMR) of 676 maternal deaths per 100,000 live births, almost no change from the 2005 DHS estimates of 673 [16]. Between 1990 and 2012, Ethiopia has shown a 67% decline in under-five mortality however, neonatal deaths declined by only 27% [8]. In both overcrowded and the underutilized facilities, the quality of maternal care, and the health of mothers and newborns, are jeopardized.

Access to and availability of medical care are both necessary but not sufficient factors to improve maternal and newborn health. In fact, they do not guarantee increased utilization of services or improved client satisfaction. Evidence is emerging that increasing the access to and utilization of facility-based maternal care alone does not necessarily translate into better maternal outcomes [17]. Poor quality of care is the most plausible explanation for this reality [13].

Now that impressive programs are being implemented in India, Ethiopia and many other high-burden countries to increase women’s access to and utilization of services (the demand side of the equation), improving health systems’ capacity to offer quality care that meets women’s needs (the supply side) is the next moral and public health imperative.

## Defining quality and measuring quality improvement

While it is difficult to define the complex and, to some extent, context-specific construct of “quality”, there are multiple definitions that provide a useful basis. For example, Goodlee (2009) defined quality care as “*clinically effective*, *safe and a good experience for the patient”*[18]. More specific to our field, Hulton et al., [19] define quality as “*the degree to which maternal health services for individuals and populations increase the likelihood of timely and appropriate treatment for the purpose of achieving desired outcomes that are both consistent with current professional knowledge and uphold basic reproductive rights*.” Quality maternal and newborn care, in this series, is defined using the Institute of Medicine (IOM) definition, i.e., care that is safe, effective, patient-centered, timely, efficient and equitable [20]. The IOM defintion of quality care is comprehensive and encompases three key components of quality: clinical (safe and effective), interpersonal (patient-centered) and contextual (timely, efficient and equitable).

Quality of care is a normative concept, in that measuring quality, or improvements in quality, demands a set of standards to guage the impact of quality improvement efforts. Quality clinical care, in maternal and newborn health, is well defined. For example, there are evidence-based guidelines for best practice in maternal and newborn care, such as those developed by the World Health Organization, or by the Royal College of Obstetrics and Gynecologists, among others. Adhering to best practice standards in clinical care would be the objective of any quality improvement effort.

The IOM defines patient-centered care as “care that is respectful of and responsive to individual patient preferences, needs, and values, and ensures that patient values guide all clinical decisions.” Defining, measuring and evaluating the quality of patient-centered care is something that the maternal health community is actively working on. The subjective and contextual nature of preferences, needs and values makes definitive “gold standards” difficult to articulate. Although beyond the scope of this paper, there is an on-going body of work to develop indicators and metrics to better define and measure the quality of patient-centered care.

As with clinical care, there are, arguably, “gold standards” for the delivery of timely, efficient and equitable care. For example, the IOM’s definitions of timely, efficient and equitable care would mandate: *no* waits or harmful delays for those who receive or give care; *no* waste of equipment, supplies, ideas or energy, and finally, *no* variation in the provision of care as a result of personal characteristics such as gender, ethnicity, geographic location, or socioeconomic status [20].

The availability of quality care varies greatly between countries and across health systems. Understanding the evidence base on interventions that facilitate “gold standard” clinical, interpersonal and contextual quality care is a critical step in improving the health and survival of women and newborns.

## Conceptual framework

Literature around quality of health care and medical practice started to emerge in the 1970s and the concept was further developed in the 1980s. By the 1990s, there were models and frameworks being developed to guide implementation, assessment and measurement of quality care, which stemmed from different conceptual understandings of the subject. They included perspective models [21] focusing on the quality of care as perceived by different constituencies: patients, healthcare providers and healthcare managers; characteristic models that listed elements and features of the care [20,22]; and systems models that put quality care, as a product of the structure of healthcare services, and the process of health care delivery [23]. However, the emergence of literature on quality of care specific to maternal and child health has been a fairly recent development.

Raven *et al*. [24] after a comprehensive analysis of the existing literature, found that a variety of perspectives have been used to approach quality of care in maternal and newborn health. Current quality improvement models include those based on assessing quality from the client’s perspective [25]; users’ experience of care and the quality of clinical care [26]; patients’ rights and providers’ needs [27,28]; appropriate intervention pathways during delivery to overcome critical delays [29], and input-output-outcome models [30-33].

Developing and applying a framework that captures most of these essential elements is critical at this juncture in the maternal and newborn health field, when access to institutional services, in particular antenatal and delivery care in clinics and hospitals, has significantly increased. Such a model will be useful in understanding the limited impact on health outcomes despite greater effort and funding to increase access to skilled care.

**Figure 2:**
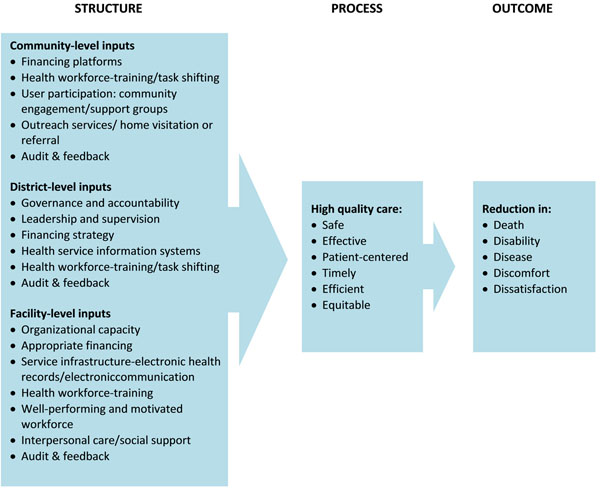
Conceptual framework

This model applies to care delivered in primary- and secondary-level facilities, with a special focus on provider behavior (both clinical and inter-personal). It combines the modified Donabedian causal chain [31,32] with other quality of care frameworks (e.g. Hulton, Maxwell, Dogba and Raven) [26,29,33,34]. The resulting framework outlines the interconnected inputs, required at different levels of health system that lead to the delivery of quality care and result in positive health outcomes (See Figure 2).

There are three key components to the Donabedian logic model: Structure, Process and Outcome [35]. In this context they have been defined as follows:

1. **Structure:** Refers to context in which healthcare is provided; Political, legal, professional and organizational resources needed to ensure that quality care is delivered, at community-, district-, and facility-levels of the health system.

2. **Processes:** Refers to whether or not good medical practices are followed, and quality care, as defined by the IOM [20] is delivered; and

3. **Outcomes:** In this framework outcomes can be divided into two domains: In addition to the traditional clinical outcomes of improved health status [35], outcomes, including positive user-experience [29], resulting in increased demand [33], and the timely utilization of healthcare services [21] have also been incorporated.

Donabedian [36] asserted that these three categories of quality measures are not independent, but are linked in an underlying framework. Good structure should promote good process and good process in turn should promote good outcomes.

The structural component of the framework includes inputs at three levels of the health system: community, district, and facility. At the community-level, the impact of outreach services, home visitation, financing platforms, community mobilization/support groups and task shifting to lay health workers were explored. Critical elements of the district-level were also considered (e.g. dimensions of governance, accountability, health workforce, infrastructure, community involvement and participation). At the facility-level, there are dimensions of leadership, health workforce, supplies, and technical capabilities. The delivery of care also addresses aspects of the work environment: provider satisfaction, provider capabilities, good environmental hygiene, evidence-based practices and user-centered care (Table 1).

**Table 1:** Definitions of structural quality of care components

**Community-level inputs** **Outreach services – Home visitation/referral:** standardized or individualized programs of additional social support provided in either home visits, during regular antenatal clinic visits, and/or by telephone on several occasions during pregnancy. **Health workforce - Task shifting:** health care workers including nurses, midwives, technicians or other lay health workers, that are lesser trained than specialized personnel but substitute for them or perform aspects of their tasks. **Health workforce – Training:** include in-service and on the job trainings, conferences, lectures, workshops, seminars, symposia, and courses. It also included additional training of outreach workers namely, lady health workers/visitors, community midwives, and community/village health workers. Clinical practice guideline implementation was also included. **Community engagement/support groups:** included formation of community support groups or formation of committee comprising of community representatives for health promotion. **District-level inputs** **Governance and accountability:** any systematic approach to ensure that services are accountable for delivering quality healthcare including audit and feedback mechanisms, medical registries, and continuous quality improvement tools. **Leadership and supervision:** provision of monitoring, guidance and feedback on matters of personal, professional and educational development in the context of the patient care **Financial strategy:** a source of motivation when an individual receives a monetary transfer which is made conditional on performing certain health related actions. **Service infrastructure-information system:** electronic health records, i.e., existence of and access to electronically retrievable health records at the point of healthcare delivery. It may also include the related training components. Electronic communication included computerized communication, telephone follow-up and counseling, interactive telephone systems, after-hours telephone access, and telephone screening **Facility-level inputs** **Well performing and motivated workforce:** included various strategies like support to manage and cope up with job, managing dual practice among healthcare workers, any form of exit interview undertaken at the time of departure from the organization. We also included interventions like changes in the organizational infrastructure, training methodologies, work environment or culture to improve the quality of care and healthcare worker performance, and audit and feedback. **Interpersonal care and social support:** included interventions provided by professionals or non-professionals aimed at improving psychological well-being. These include various supportive interventions delivered in home visits, antenatal clinics or by telephone. **Safety culture:** any interventions to enhance the safety of healthcare workers in healthcare environment. These included hand hygiene, interventions to reduce medication errors and influenza vaccination administered to health care professionals working in facility set-ups. **Staffing models:** as the organizational interventions for nursing care like staffing levels, skill mix, qualification or grade mix, staff-patient ratios etc. We also included intervention to improve collaboration between two or more health and/or social care professionals.

High quality care is a necessary process for improved health outcomes [20]. Improvements in any of these systematic or process dimensions of quality are likely to result in better maternal and newborn health outcomes: reductions in death, disease, disability, discomfort and dissatisfaction with the care provided.

This quality of care framework was developed as an easy-to-use and conceptual guide to understand the drivers of quality in facility-based maternal care. This framework adds dimensions to existing Donbedian frameworks that have been used to assess quality of care. For example, Dogba *et al.*, examined the evidence of material, human and organizational structures, on clinical and interpersonal processes of care, and the provision of basic emergency obstetric and newborn care [33]. The conceptual framework presented here utilizes the same underlying Donabedian framework, but disaggregates structrual elements into three distinct health-system levels. Interventions aimed at improving inputs at any of these levels would not only advance the clinical provision of care, but would also result in greater patient satisfaction with the services provided. Women’s satisfaction with the care they receive may feed back into higher levels of facility utilization.

A key attribute of this framework is that it is flexible enough to meet the context-specific needs of the care setting. The split of structural components into community-, district-, and facility-level inputs allows the framework to be used by decision-makers at each of these three levels. It also allows for a greater understanding of the interplay between each of these levels, which are inextricably linked but can also, independently, improve the delivery of quality care. For example, financing strategies at the district-level may directly impact the process of care, but if the facility-level input of a well-performing and motivated workforce is not ensured, any direct improvement in the quality of care may be jeopardized. The logic model of this framework allows policy makers, program implementers and providers to examine the causal and interconnected pathways between community-, district- and facility- based interventions, on the quality of care provided.

A better understanding of the interplay between community-, district-, and facility-level structural factors that facilitate the delivery of high quality maternal health services is critical. Ultimately this will lead to the successful implementation of interventions to improve maternal and newborn outcomes. This framework offers a way to examine the pathways and connections between these different levels in the provision of quality maternal health services.

## Why facilities, why this time frame?

The Maternal Health Task Force (MHTF), the flagship program of the Women and Health Initiative at the Harvard School of Public Health, focuses its work on improving and measuring the quality of institutional care provided to women once they have accessed the health system. The work of the MHTF focuses on the third trimester, labor and delivery, as well as the immediate postpartum period, the most hazardous time for women and newborns. During this critical period, women and newborns face multiple risks: placenta previa and other causes of pre and post-partum hemorrhage, pre-eclampsia/eclampsia, dystocia, infection, and other conditions that are most relevant in this timeframe. These conditions directly impact maternal and newborn survival. Effective detection and management of these complications require facility-based, skilled care.

In this collection, findings from systematic reviews on the impact of various approaches to improve the quality of care for women and newborns was assessed and summarized. The focus of this review was to identify the evidence base, and information gaps, on approaches that enable health providers to adopt and implement patient-centered, evidence-based interventions to improve the quality of care during childbirth and the immediate postpartum period.

The findings from this review will be used to broaden the evidence base for interventions at multiple levels of the health system and to identify knowledge gaps that represent priority research questions. Following this introductory paper, three additional papers present the findings of systematic reviews of community-, district- and facility-level interventions aimed at improving the quality of maternal health care. In the final paper, the findings of the review will be comprehensively discussed, and recommendations and next steps will be proposed. Ideally, this framework will continue to evolve as evidence emerges on innovative ways to improve and measure the quality of facility-based maternal health care.

## Methods

We considered all available systematic reviews on the approaches at various levels of the Quality of Care (QoC) framework (Figure 2), which are directly applicable to women and newborn health. We also included reviews with a focus on the interventions directed towards the frontline workers’ implementation of known interventions with its impact on maternal newborn health. Our priority was to select existing Cochrane and non-Cochrane reviews of randomized or non-randomized controlled trials, which fully or partly address the interventions for improving the maternal-newborn health domain; we have reported the impacts on general health outcomes as reported by the reviewers. These outcomes include screening and use of mammograms, although not directly related to maternal-newborn health but broadly related to quality of care.

## Inclusion criteria

Systematic reviews with approaches having an impact on the frontline workers’ implementation of known interventions were reviewed in the following domains:

a. Community-level inputs

i. Financing platforms

ii. Health workforce-training/task shifting

iii. User participation: community engagement/support groups

iv. Outreach services/home visitation/referral

b. District-level inputs

i. Governance and accountability

ii. Leadership and supervision

iii. Financing strategy

iv. Service infrastructure

v. Health information systems

vi. Health workforce-training/task shifting

vii. Audit and feedback

c. Facility-level inputs

i. Organizational capacity

ii. Appropriate financing

iii. Service infrastructure-electronic health records/electronic communication

iv. Health workforce-training

v. Well-performing and motivated workforce

vi. Interpersonal care and social support

## Search strategy

All available systematic reviews for the impact of quality of care interventions published by May 2013 were reviewed. The following sources of information were used to search literature for review:

1. All available electronic references libraries of indexed medical journals and analytical reviews

2. Electronic reference libraries of non-indexed medical Journals

3. Non-indexed journals not available in electronic libraries

4. Pertinent books, monographs, and theses identified through electronic or hand searching

5. Project documents and reports

The following principal sources of electronic reference libraries were searched to access the available data: The Cochrane Library, Medline, PubMed, Popline, LILACS, CINAHL, EMBASE, World Bank's JOLIS search engine, CAB Abstracts, British Library for Development Studies BLDS at IDS, the World Health Organization regional databases as well as the IDEAS database of unpublished working papers, Google and Google Scholar. Detailed examination of cross-references and bibliographies of available data and publications to identify additional sources of information was also done. A broad search strategy was used that included a combination of appropriate key words, medical subject heading (MeSH) and free text terms. Search algorithms are added within the individual components.

## Type of outcomes

The following is an illustrative listing of outcomes of interest:

• Healthcare outcomes as assessed by a variety of measures. These included mortality; morbidity; physiological measures; and participants’ self-reports of symptom resolution, quality of life, or patient self-esteem.

• Service coverage.

• Health behaviors, such as adherence of clients/patients to medication or dietary supplements.

• Harms or adverse effects

• Recipient satisfaction with care

• Utilization of services

• Costs

• Providers’ adoption of evidence-based interventions and compliance with desired practice

• Other provider-related aspects of quality of care

## Data extraction and analysis

The project team set up a triage process with standardized criteria for evaluating outputs from the search strategy and primary screening. Following an agreement on the search strategy, the abstracts (and the full sources where abstracts were not available) were screened by two abstractors to identify studies adhering to our objectives. Any disagreements on selection of studies between these two primary abstractors were resolved by the third reviewer. After retrieval of full texts of all the reviews that met the inclusion/exclusion criteria, each review was double data abstracted into a standardized form. Information was extracted on the following criteria:

1. Characteristics of included reviews - description of each review included brief description of methods, participants, interventions, outcomes and note on specific issue (if any);

2. Extraction of measurement of treatment effects;

3. Methodological determinants;

4. Risk of bias tool; and

5. Quality assessment.

Available systematic reviews were assessed for quality using the AMSTAR criteria (Assessment of the methodological quality of systematic reviews) (Table 2) [37]. We resolved any disagreement by discussion and the final decision was taken by consensus within the team.

**Table 2:** Assessing methodological quality of systematic reviews (AMSTAR) criteria

**1. Was *****a priori***** design provided?** The research question and inclusion criteria should be established before the conduct of the review.
**2. Was there duplicate study selection and data extraction?** There should be at least two independent data extractors and a consensus procedure for disagreements should be in place.

**3. Was a comprehensive literature search performed?** At least two electronic sources should be searched. The report must include years and databases used (e.g. Central, EMBASE, and MEDLINE). Key words and/or MESH terms must be stated and where feasible the search strategy should be provided. All searches should be supplemented by consulting current contents, reviews, textbooks, specialized registers, or experts in the particular field of study, and by reviewing the references in the studies found.

**4. Was the status of publication (i.e. grey literature) used as an inclusion criterion?** The authors should state that they searched for reports regardless of their publication type. The authors should state whether or not they excluded any reports (from the systematic review), based on their publication status, language etc.

**5. Was a list of studies (included and excluded) provided?** A list of included and excluded studies should be provided.

**6. Were the characteristics of the included studies provided?** In an aggregated form such as a table, data from the original studies should be provided on the participants, interventions and outcomes. The ranges of characteristics in all the studies analyzed e.g. age, race, sex, relevant socioeconomic data, disease status, duration, severity, or other diseases should be reported

**7. Was the scientific quality of the included studies assessed and documented?** A priori' methods of assessment should be provided (e.g., for effectiveness studies if the author(s) chose to include only randomised, double-blind, placebo controlled studies, or allocation concealment as inclusion criteria); for other types of studies alternative items will be relevant.

**8. Was the scientific quality of the included studies used appropriately in formulating conclusions?** The results of the methodological rigor and scientific quality should be considered in the analysis and the conclusions of the review, and explicitly stated in formulating recommendations

**9. Were the methods used to combine the findings of studies appropriate?** For the pooled results, a test should be done to ensure the studies were combinable, to assess their homogeneity (i.e. Chi-squared test for homogeneity, I^2^). If heterogeneity exists a random effects model should be used and/or the clinical appropriateness of combining should be taken into consideration (i.e. is it sensible to combine?)

**10. Was the likelihood of publication bias assessed?** An assessment of publication bias should include a combination of graphical aids (e.g., funnel plot, other available tests) and/or statistical tests (e.g., Egger regression test).

**11. Was the conflict of interest included?** Potential sources of support should be clearly acknowledged in both the systematic review and the included studies.

Responses to each: Yes, No, Can’t Answer, Not Applicable.

## Strengths and limitations

This research was based on a review of systematic reviews of the evidence, on improving the quality of care at the community-, district- and facility-level. Systematic reviews and meta-analysis have become increasingly popular in evidence-based healthcare over the past two decades [38] although these have been evaluated for reliability since quite a long time [39-42]. Rigorous and transparent systematic reviews are recognized internationally as a credible source for evidence of effects and as the basis for evidence-informed policy and decisions [38,43,44]. Overview of systematic reviews offers some important advantages: 1) it builds on the conclusions of rigorous reviews of multiple quality intervention studies in different settings; 2) it avoids duplication of work done by other researchers; 3) it allows for a much faster review. The methodology adopted has some limitations: 1) interventions on which primary data exists, but which have not been covered by a systematic review, will not have been included; 2) it relies on review authors’ characterizations of the findings rather than on individual studies and therefore may be affected by selective reporting biases; 3) it will miss studies not taken up by included reviews.

With this understanding of the conceptual framework and methodology, we aimed to evaluate the effectiveness of interventions for improving the quality of care at the community-, district- and facility-level for maternal newborn health through existing systematic reviews in the following papers [45-47].

## Competing interests

We do not have any financial or non-financial competing interests for this review.

## Author contributions

All authors contributed to the writing of the paper.

## Peer review

Peer review reports are included in Additional file 1.

## Supplementary Material

Additional file 1 Click here for file

## Declarations

This article has been published as part of *Reproductive Health* Volume 11 Supplement 2, 2014: Quality of Care in Maternal and Child Health. The full contents of the supplement are available online at http://www.reproductive-health-journal.com/supplements/11/S2.
