# Supplementary material for: Approaches to improve the quality of maternal and newborn health care: an overview of the evidence
Source: Reprod Health. 2014 Sep 4;11(Suppl 2):S1. doi: 10.1186/1742-4755-11-S2-S1 (PMC4160919; doi:10.1186/1742-4755-11-S2-S1)
Supplement: Additional file 1 [file 1742-4755-11-S2-S1-S1.pdf]

Referee's comments to the authors– this sheet <u>WILL</u> be seen by the author(s) and published alongside the article

Article ref no.	
Title	Evidence from community level inputs to improve quality maternal and newborn health: Interventions and findings
Author(s)	Zohra S lassi; Jai K. Das; Rehana A Salam & Zulfiqar A Bhutta
Referee's name	J. Shea

The series is certainly relevant and important. The foundational premise of the manuscript is significant as it foregrounds the need for improved access to health services as well as the need for high quality health care.

<u>Abstract</u>: The question/objective is not articulated.

<u>Introduction:</u> The first paragraph contains information that highlights the problem. I suggest inserting a topic/introductory sentence that includes maternal and neonatal mortality.
"Among women who avoid maternal death …" – can be revised.
"and the trend is expected to continue as the under five deaths reduce disproportionately"(p.3) – what does this mean?
The definition of "quality in health care" does not adequately feature current debates and contentions about quality of care.
The content of the 1st paragraph on page 5 is good.
The data in the ***Introductory*** section are not presented systematically or well, and the flow between paragraphs is not effective, which hampers the overall coherence of the paper.

<u>Conceptual Framework</u>: The Conceptual Framework presented on page 20 appears to follow an abridged Logic model and encompasses salient aspects of quality care. It would be worth considering streamlining points iv and v in the *District Level Inputs* component. One example would be Health Information Systems rather than *Service-Infrastructure-Electronic health records/electronic communication*. The framework will benefit from strategic revision that takes into account current terminology, like the Health Workforce and Community Engagement, for example. Regarding quality health care, it is not only the training component or task-shifting that is key; it is unclear why these two features are emphasized.

<u>Why facilities, why this time frame?:</u> This paragraph is good:" This quality of care framework was developed to be an easy-to-use guide to understand the drivers of quality in facility-based maternal care. A key attribute of this framework is that it is also flexible enough to meet the context-specific needs of the care setting. The split of structural components into district-, facility-, and community-level inputs allows the framework to be used by officials at each of these three levels, and will allow for a greater understanding of the interplay between each of these levels."

Panel 1: Definition of quality care components:-
The Leadership and Supervision definition does not adequately incorporate a leadership definition.
Service Infrastructure-Information System – does this relate only to patient-related information?

Search Strategy: The search strategy for systematic reviews is explained in detail. It appears that the authors chose approaches that have an impact on frontline workers' implementation of interventions, and included search terms or strings embedded in the conceptual framework. It is important to note that Cochrane Systematic Reviews include only Randomised Controlled Trials (RCTs) and do not include a wide variety of other research designs, like mixed method studies. General information about the study designs included in reviews other than Cochrane Systematic Reviews is recommended.

The manuscript suffers from a lack of attention to scholarly writing. There are several language edits required throughout the paper, starting with the Abstract. For example:

- the first sentence of the Introduction reads: " Although, important progress has been made in reducing maternal mortality in a number of countries (1,2,3], globally an estimated 273,500 maternal deaths occurred in 2011[3]." This sentence is clumsy, poorly structured, and referenced inappropriately.
- Evidence have proven that the quality of care received by women and newborns …." (p.2) - revise
- Southern Asia also needs to address their neonatal mortality: with neonatal deaths accounting for more than half of under-five deaths in 2011, with almost 30% of the global neonatal deaths occurring in India alone [10].- (p.3) this sentence needs revision.
- A number of evidence-based interventions to prevent and successfully manage all major causes of maternal morbidity and mortality, including good nutrition, access to contraception, skilled attendance at delivery, and emergency obstetric care are available[11], although the delivery and distribution of these services is unfortunately compromised [11,12].- (p.3)the sentence is too long.
- Hulton et al [4] – (p.4) Hulton et al.
- Althabe et al [13} – (p.4) Althabe et al.
- Raven et al [32}- (p.5) Raven et al.
- such an explanation might illuminate why there are such minimal improvements in maternal health.. (p.6) – delete the word "such"

The manuscript needs complete language/editing revision.

Approaches to Improve Quality of Maternal and Newborn Health Care: An Overview of the Evidence

Paper 1 of 5 paper-series review: Introduction, Conceptual Framework and Methods

Anne Austin[1], Ana Langer[1], Rehana A Salam[2], Zohra S Lassi[2], Jai K Das[2], Zulfiqar A Bhutta[23]*

1. Harvard School of Public Health, Boston, USA
2. Division of Women & Child Health, The Aga Khan University, Karachi, Pakistan
3. Program for Global Pediatric Research, Hospital For Sick Children, Toronto

Emails
AA: aaustin@hsph.harvard.edu
RAS: rehana.salam@aku.edu
JKD: jai.das@aku.edu
ZSL: zohra.lassi@aku.edu
AL: alanger@hsph.harvard.edu

*Corresponding author
Zulfiqar A. Bhutta
MBBS, FRCP, FRCPCH, FCPS, PhD
HuseinLaljiDewrajProfessor&Head
Division of Women & Child Health
The Aga Khan University
Karachi 74800
Pakistan

Email: zulfiqar.bhutta@aku.edu

Abstract:

Millions of women and children die from preventable causes and almost all of these deaths occur in low- and middle-income countries. Yet, global progress in limiting these has been slow and the burden is particularly striking in the regions where they have lowest access to and application of facility-based services for childbirth and newborn care. Evidence have proven that the quality of care received by women and newborns predominantly in marginalised group is poor, and is the major contributing factor for higher rates of morbidity and mortality. In this series of five papers, we aim to systematically assess and summarize information from relevant systematic reviews on the impacts of various approaches to improve the quality of care for women and newborns. This paper will be an introductory paper detailing the conceptual framework and methodology used in the following three papers on the findings of systematic reviews of community, facility and district level interventions aimed at improving the quality of maternal health care while the last paper will discuss the findings of the review, and propose recommendations.

Introduction

Although, important progress has been made in reducing maternal mortality in a number of countries [1,2,3], globally an estimated 273,500 maternal deaths occurred in 2011[3]. Sub-Saharan Africa (56%) and Southern Asia (29%) accounted for 85% of the global burden [1], while at country level, two countries contributed to a third of global maternal deaths: India at 19% (56 000) and Nigeria at 14% (40 000) (**Figure 1**). Among women who avoid maternal death, approximately 10 million suffer from complications related to pregnancy and childbirth [5,6], and over 80% of these deaths could be prevented or avoided through timely interventions proven to be effective and affordable [7,8,9].

On the other hand, global neonatal mortality has declined by 32%, from 32 deaths per 1,000 live births in 1990 to 22 in 2011. Although these declines are slower than those in the mortality rate for older children, the share of neonatal deaths among under-five deaths increased from about 36% in 1990 to about 43% in 2011, and the trend is expected to continue as the under five deaths reduce disproportionately. From the last available estimates Sub-Saharan Africa, which accounts for 38% of global neonatal deaths, has the highest neonatal mortality rate (34 deaths per 1,000 live births in 2011) and is among the regions showing least progress over the last two decades. Southern Asia also needs to address their neonatal mortality: with neonatal deaths accounting for more than half of under-five deaths in 2011, with almost 30% of the global neonatal deaths occurring in India alone [10].

All in all, millions of women and children die from preventable causes and almost all of these deaths (99%) occur in low- and middle-income countries (LMIC). Yet, global progress in limiting these has been slow and the burden is particularly striking in the regions where they have lowest access to and application of facility-based services for childbirth and newborn care. A number of evidence-based interventions to prevent and successfully manage all major causes of maternal morbidity and mortality, including good nutrition, access to contraception, skilled attendance at delivery, and emergency obstetric care are available[11], although the delivery and distribution of these services is unfortunately

compromised [11,12]. Therefore, concern for these deprived populations demands improved efforts to speed up and scale up the implementation of effective interventions through increased access to and utilization of better quality care. Evidence have proven that the quality of care received by women and newborns predominantly in marginalised group is poor, and is the major contributing factor for higher rates of morbidity and mortality [13,14,15].

While it is dificult to define “quality” and gauge the “quality of care”, there are some simple definitions, such as care that is “*clinically effective, safe and a good experience for the patient”*[16]. A more comprehensive description refers to quality of care in terms of meeting standards of care that are safe, effective, patient centered, timely, efficient and equitable[17]. More specifically to our field, Hulton et al[4] define quality of care as the degree to which maternal health services for individuals and populations increase the likelihood of timely and appropriate treatment for the purpose of achieving desired outcomes that are both consistent with current professional knowledge and uphold basic reproductive rights.

The interventions to improve the QoC are situational, tailored and context specific. Several factors have been modeled for improved delivery of care that are applicable to most settings. Hulton et al [4] identified two equally important and parallel lists of interventions, which comprise of provision and experience of care (**Figure 2)**.

Systematic reviews have been conducted for the various factors involved in improving the quality of care. For example, Althabe et al [13] reviewed educational, financial and organizational interventions for improving quality of care. A similar set of interventions has been included in The Cochrane Effective Practice and Organisation of Care (EPOC). Several other systematic reviews looked at continuing education programs and educational strategies for quality improvement in health care [18,19,20,21]. Also, there are systematic reviews on organisational [22], financial [23,24] and regulatory interventions [25]. Audit and feedback mechanisms for improving professional practice have also been evaluated [26,27]. Likewise, mass-media and patient-mediated interventions may also impact professional practice. These reviews have pointed towards the potential benefits of these strategies to

improve health care practice. However, scaling up and sustainability may be difficult to achieve and need careful consideration. While interventions for improving quality of care should be context specific, important lessons can be learned in evaluating interventions in different settings specifically for maternal and newborn health. Targeted and collaboratively designed approaches to intervention delivery need to be highlighted to improve the health outcomes of these two critical population groups, especially in low resource settings.

Conceptual Framework

Literature around quality of healthcare and medical practice started to emerge in the 70s and the concept developed to some extent in the 80s. By the 90s, there were models and frameworks being developed for implementing, assessing and measuring quality care which stemmed from different conceptual understandings of the subject. They included perspective models [28] focusing on the quality of care as perceived from different perspectives – that of patients, healthcare providers and healthcare managers; Characteristic models that listed elements and characteristics of the care[29,30]; and systems models that defined quality of care as a product of structure of healthcare services, quality of the process and the quality of the outcome[31]. However, the emergence of literature on quality of care specific to maternal and child health has been a fairly recent development. Raven et al [32] after a comprehensive analysis of the existing literature and identifying several models, found that a variety of perspectives can have been used to approach quality of care in maternal and newborn health.

The current models of quality of maternal healthcare range from models based on assessing quality from the client's perspective (perspective model) [33]; models based on the users' experience of care and the quality of the provision of care [34]; models based on patients' rights and providers' needs; models based on appropriate-intervention pathways during delivery to overcome critical delays, models based on women's progression through delivery (PAHO), perspective based models (International Council on Management of Population Programs), and input-output-outcome based models.

Developing and applying a model that captures most of these essential elements is critical at this juncture of the maternal and newborn health field, when access to institutional

services, in particular antenatal and delivery care in clinics and hospitals, has significantly increased. Access and availability to medical care are necessary but not sufficient factors to improve maternal health outcomes. In fact, they do not guarantee the utilization of services or improved client satisfaction, and evidence is emerging that increasing the access to and utilization of facility-based maternal care, alone, does not necessarily translate into better maternal outcomes[35].

For example, in India and Ethiopia, two countries that account for one-fifth of global maternal deaths, large investments in infrastructure and provider training have not yet yielded the expected dramatic improvements in maternal health.[36] Maternal mortality ratios have not decreased uniformly and in some areas have remained stubbornly high. In India, the conditional cash transfer program, Janani Suraksha Yojana (JSY), has resulted in an increased number of institutional births, but it is unclear whether this has actually resulted in improved maternal health outcomes.[37]

Ethiopia presents a different scenario: historically, the Health Sector Development programs have focused on improving the facility infrastructure, training health care providers and promoting referrals to health facilities for birth. Despite these efforts, only 10% of Ethiopian women have facility-based births [38]. In both the overcrowded and the underutilized facilities, the quality of maternal care is jeopardized. A better understanding of the district, facility and community level factors that facilitate high quality maternal health services, and the pathways and connections between these issues, is needed. Such an explanation might illuminate why there are such minimal improvements in maternal health despite greater effort and funding to increase access to skilled care.

Now that impressive programs are being implemented in India, Ethiopia and other high-burden countries to increase women's access to and utilization of services (the demand side of the equation), improving health systems' capacity to offer quality care that meets women's needs (the supply side) is the next moral and public health imperative.

Why facilities, why this time frame?

The Maternal Health Task Force (MHTF), the flagship program of the Women and Health Initiative at the Harvard School of Public Health, focuses its work on improving and measuring the quality of institutional care provided to women once they have accessed the

health system. The work of the MHTF focuses on the third trimester as well as the immediate postpartum period. Although the latter is the most hazardous to women, the third trimester of pregnancy is also a the period of higher occurrence of pregnancy complications, including placenta previa and other causes of hemorrhage, pre-eclampsia and eclampsia, and dystocia, among other conditions. Effective detection and management of these complications require facility-based, skilled care.

Comment [FA1]: Please review if you agree with the suggestion.

Our model applies to care delivered in primary and secondary-level facilities, with a special focus on provider behavior (both clinical and inter-personal). The structural component of the framework includes inputs at three levels: district, facility, and community. Critical elements of the district level are included as contextual factors (e.g. dimensions of governance, human resources, infrastructure, community involvement and participation). At the facility level, there are dimensions of leadership, human resources, supplies and technical capabilities. At the community level, outreach services, home visitation, financing platforms, community mobilization/support groups and task shifting to lay health workers are included. The delivery of care also includes aspects of the work environment, provider satisfaction, provider capabilities, good environmental hygiene, evidence-based practices and user-centered care. (**Figure 3)**

High quality care is defined in accordance with the Institute of Medicine's definition: care that is safe, timely, effective, efficient, equitable and responsive to patients' needs and preferences [30]. Improvements in any of these dimensions of quality are likely to result in the increased possibility of desired maternal and newborn health outcomes: reductions in death, disease, disability, discomfort and dissatisfaction with the care provided and an improved health outcome. (**Refer to Panel 1 for definitions)**

This quality of care framework was developed to be an easy-to-use guide to understand the drivers of quality in facility-based maternal care. A key attribute of this framework is that it is also flexible enough to meet the context-specific needs of the care setting. The split of structural components into district-, facility-, and community-level inputs allows the

framework to be used by officials at each of these three levels, and will allow for a greater understanding of the interplay between each of these levels.

In this collection, we aim to systematically assess and summarize information from relevant literature and systematic reviews on the impacts of various approaches to improve the quality of care for women and newborns. The focus of this review was specifically on approaches that enable frontline workers (trained health providers either in the community or in a facility) to adopt and implement patient-centered, evidence-based interventions to improve the quality of care during childbirth and immediate postpartum period.

In the following papers we will present and discuss the results of systematic reviews of the existing evidence, which we will use to populate the quality of care framework and to identify knowledge gaps that represent priority research questions. After this introductory paper, the following three present the findings of systematic reviews of community, facility and district level interventions aimed at improving the quality of maternal health care. In the last paper, we comprehensively discuss the findings of the review, and propose recommendations and next steps. Ideally, this framework will evolve as evidence emerges on innovative ways to improve the quality of facility-based maternal health care. Equally importantly, as gaps are identified, it is hoped that a clear research agenda will emerge.

PANEL 1: DEFINITION OF QUALITY OF CARE COMPONENTS

- ***District Level Inputs***

Governance and Accountability: any systematic approach to ensure that services are accountable for delivering quality healthcare including audit and feedback mechanisms, medical registries, and continuous quality improvement tools.

Leadership and Supervision: provision of monitoring, guidance and feedback on matters of personal, professional and educational development in the context of the patient care

Financial Strategy: a source of motivation when an individual receives a monetary transfer which is made conditional on performing certain health related actions.

Service Infrastructure-Information System: electronic health records as provision and access to electronically retrievable health records at the point of healthcare delivery. It may also include the related training components. Electronic communication included computerized communication, telephone follow-up and counseling, interactive telephone systems, after-hours telephone access, and telephone screening

- ***Community Level Inputs***

Outreach services-Home Visitation/Referral: standardized or individualized programs of additional social support provided in either home visits, during regular antenatal clinic visits, and/or by telephone on several occasions during pregnancy.

Human Resource-Task Shifting: standardized or individualized programs of additional social support provided in either home visits, during regular antenatal clinic visits, and/or by telephone on several occasions during pregnancy.

Human Resources-Training: include in-service trainings, conferences, lectures, workshops, seminars, symposia, and courses. It also included additional training of outreach workers namely, lady health workers/visitors, community midwives, and community/village health workers. Clinical practice guideline implementation was also included.

Community Mobilization/Support Groups: included formation of community support groups or formation of committee comprising of community representatives for health promotion.

- ***Facility Level Inputs***

Well Performing and Motivated Workforce: included various strategies like support to manage and cope up with job, managing dual practice among healthcare workers, any form of exit interview undertaken at the time of departure from the organization. We also included interventions like changes in the organizational infrastructure, work environment or culture to improve the quality of care and healthcare worker performance.

Interpersonal care and social support: included interventions provided by professionals or non-professionals aimed at improving psychological well-being. These include various supportive interventions delivered in home visits, antenatal clinics or by telephone.

Safety Culture: any interventions to enhance the safety of healthcare workers in healthcare environment. These included hand hygiene, interventions to reduce medication errors and influenza vaccination administered to health care professionals working in facility set-ups.

[illegible]

Methods

We considered all available systematic reviews on the approaches at various levels of the QoC framework (**Figure 3**), which are directly applicable to women and newborn health. We also included reviews with a focus on the interventions directed towards the frontline workers' implementation of known interventions with its impact on maternal newborn health. Our priority was to select existing Cochrane and non-Cochrane reviews of randomized or non-randomized controlled trials, which fully or partly address the interventions for improving maternal newborn health domain; we have reported the impacts on general health outcomes as reported by the reviewers. These also include outcomes like screening and use of mammogram, although not directly related to maternal newborn health but broadly related to quality of care.

Inclusion criteria: We included systematic reviews with approaches having an impact on the frontline workers' implementation of known interventions in the following domains:

a. **District Level Inputs**
    i. Governance and Accountability
    ii. Leadership and Supervision
    iii. Financing Strategy
    iv. Service Infrastructure-Electronic health records/electronic communication
    v. Human Resources-Training/Task shifting
b. **Community level Inputs**
    i. Financing platforms
    ii. Human Resource-Training/Task shifting
    iii. User participation: Community mobilization/ support groups
    iv. Outreach services/ home visitation/ referral
c. **Facility Level Inputs**
    i. Organizational Capacity
    ii. Appropriate Financing
    iii. Service Infrastructure-Electronic health records/electronic communication
    iv. Human Resource-Training
    v. Well-performing and Motivated Workforce
    vi. Interpersonal care and social support

Search strategy: All available evidence for the impact of quality of care interventions was systematically analyzed. The following sources of information were used to search literature for review:

Comment [FA2]: Please include time period.

1. All available electronic references libraries of indexed medical journals and analytical reviews
2. Electronic reference libraries of non-indexed medical Journals
3. Non-indexed journals not available in electronic libraries
4. Pertinent books, monographs, and theses identified through electronic or hand searching
5. Project documents and reports

The following principal sources of electronic reference libraries were searched to access the available data: The Cochrane Library, Medline, PubMed, Popline, LILACS, CINAHL, EMBASE, World Bank's JOLIS search engine, CAB Abstracts, British Library for Development Studies BLDS at IDS, the World Health Organization (WHO) regional databases as well as the IDEAS database of unpublished working papers, Google and Google Scholar. Detailed examination of cross-references and bibliographies of available data and publications to identify additional sources of information will also be performed. In particular, this search was also extended to review the gray literature in non-indexed and non-electronic sources. A broad search strategy was used that included a combination of appropriate key words, MESH and free text terms. Search algorithms are added within the individual components.

Types of outcomes: The following is an illustrative listing of outcomes of interest:

- Healthcare outcomes as assessed by a variety of measures. These included mortality; morbidity; physiological measures; and participants' self-reports of symptom resolution, quality of life, or patient self-esteem.
- Service coverage.
- Health behaviors, such as adherence of clients/patients to medication or dietary supplements.
- Harms or adverse effects
- Recipient satisfaction with care
- Utilization of services

- Costs
- Providers' adoption of evidence-based interventions and all other provider-related aspects of quality of care

Data extraction and analysis: The project team set up a triage process with standardized criteria for evaluating outputs from the search strategy and primary screening. Following an agreement on the search strategy, the abstracts (and the full sources where abstracts not available) were screened by two abstractors to identify studies adhering to our objectives. Any disagreements on selection of studies between these two primary abstractors were resolved by the third reviewer. After retrieval of the full texts of all the reviews/studies that met the inclusion/exclusion criteria, each review/study was double data abstracted into a standardized form. Information was extracted on the following criteria:

1. Characteristics of included reviews - description of each review included brief description of methods, participants, interventions, outcomes and note on specific issue (if any);
2. Extraction of measurement of treatment effects;
3. Methodological issues;
4. Risk of bias tool; and
5. Quality assessment of included reviews.

Available systematic reviews were assessed for quality using the AMSTAR criteria (Assessment of the methodological quality of systematic reviews) (Appendix 1) [39]. We resolved any disagreement by discussion and the final decision was taken by consensus within the team.

The next part of this 5 series review will focus on interventions and findings of District Level Inputs on QoC for women and newborns.

Comment [FA3]: However the series order seem to include Community interventions as the second paper

Competing Interests:

We do not have any financial or non-financial competing interests for this review.

Authors Contribution:

Dr. Zulfiqar A Bhutta (ZAB), Anne Austin (AA), Ana Langer (AL) designed and co-ordinated the review. Rehana A Salam (RAS), Zohra S Lassi (ZSL) and Jai K Das (JKD) were responsible for: data collection, screening the search results, screening retrieved papers against inclusion criteria, appraising quality of papers, abstracting and interpreting data. ZAB, AA and AL critically reviewed and modified the manuscript.

Acknowledgment:

This work was supported by a grant from the Maternal health Task Force (MHTF) at the Harvard School of Public Health. We would like to acknowledge Waleed Zahid who helped us in the search and abstraction of data.

References

1. WHO, UNICEF, UNFPA, estimates TWB (2012) WHO: Trends in maternal mortality: 1990 to 2010. http://wwwunfpaorg/webdav/site/global/shared/documents/publications/2012/Trends_in_maternal_mortality_A4-1pdf.
2. Hogan MC, Foreman KJ, Naghavi M, Ahn SY, Wang M, et al. (2010) Maternal mortality for 181 countries, 1980-2008: a systematic analysis of progress towards Millennium Development Goal 5. Lancet 375: 1609-1623.
3. Lozano R, Wang H, Foreman KJ, Rajaratnam JK, Naghavi M, et al. (2011) Progress towards Millennium Development Goals 4 and 5 on maternal and child mortality: an updated systematic analysis. Lancet 378: 1139-1165.
4. Hulton LA, Mathews Z, Stones RW (2000) A framework for the evalaution of quality of care in maternity services Southampton: University of Southampton.
5. Filippi V, Ronsmans C, Campbell OMR, Graham WJ, Mills A, et al. (2006) Maternal survival 5 - Maternal health in poor countries: the broader context and a call for action. Lancet 368: 1535-1541.
6. Ronsmans C GW (2006) Maternal survival 1 - Maternal mortality: who, when, where, and why. Lancet 368: 1189-1200.
7. WHO (2004) Beyond the Numbers: Reviewing Maternal Deaths and Complications to Make Pregnancy Safer. Geneva: Department of Reproductive Health and Research, World Health Organisation.
8. De Brouwere V, Tonglet R, Van Lerberghe W (1998) Strategies for reducing maternal mortality in developing countries: what can we learn from the history of the industrialized West? . Trop Med Int Health 3: 771-782.
9. Khan KS, Wojdyla D, Say L, Gulmezoglu AM, Van Look PF (2006) WHO analysis of causes of maternal death: a systematic review. Lancet 367: 1066-1074.
10. UNICEF, WHO, Bank W, Division UP (2012) Levels and trends in child mortality.
11. Friberg IK, Kinney MV, Lawn JE, Kerber KJ, Odubanjo MO, et al. (2010) Sub-Saharan Africa's mothers, newborns, and children: how many lives could be saved with targeted health interventions? PLoS Med 7: e1000295.
12. (2010) Maternal health: time to deliver. PLoS Med 7: e1000300.
13. Althabe F, Bergel E, Cafferata ML, Gibbons L, Ciapponi A, et al. (2008) Strategies for improving the quality of health care in maternal and child health in low- and middle-income countries: an overview of systematic reviews. Paediatr Perinat Epidemiol 22 Suppl 1: 42-60.
14. van den Broek NR, Graham WJ (2009) Quality of care for maternal and newborn health: the neglected agenda. BJOG 116: 18-21.
15. Wagaarachchi PT, Fernando L (2002) Trends in maternal mortality and assessment of substandard care in a tertiary care hospital. Eur J Obstet Gynecol Reprod Biol 101: 36-40.
16. Godlee F (2009) Effective, safe and a good patient experience. BMJ 339: b4346.
17. Buchan H (2010) World Health Organization. Sharing Knowledge for Action on Maternal, Newborn and Child Health. Geneva The Partnership for Maternal, Newborn and Child Health, World Health Organization

18. Farmer AP, Legare F, Turcot L, Grimshaw J, Harvey E, et al. (2008) Printed educational materials: effects on professional practice and health care outcomes. Cochrane Database Syst Rev 3: CD004398.
19. McGowan J, Grad R, Pluye P, Hannes K, Deane K, et al. (2009) Electronic retrieval of health information by healthcare providers to improve practice and patient care. Cochrane Database Syst Rev 3: Issue 3. Art. No.: CD004749.
20. Forsetlund L, Bjorndal A, Rashidian A, Jamtvedt G, O'Brien MA, et al. (2009) Continuing education meetings and workshops: effects on professional practice and health care outcomes. Cochrane Database Syst Rev 2: CD003030.
21. O'Brien MA, Rogers S, Jamtvedt G, Oxman AD, Odgaard-Jensen J, et al. (2007) Educational outreach visits: effects on professional practice and health care outcomes. Cochrane Database Syst Rev 4: Issue 4. Art. No.: CD000409.
22. Dudley L, Garner P (2011) Strategies for integrating primary health services in low- and middle-income countries at the point of delivery. Cochrane Database Syst Rev 7: Issue 7. Art. No.: CD003318.
23. Gosden T, Forland F, Kristiansen IS, Sutton M, Leese B, et al. (2000) Capitation, salary, fee-for-service and mixed systems of payment: effects on the behaviour of primary care physicians. Cochrane Database Syst Rev Issue 3. Art. No.: CD002215: CD002215.
24. Giuffrida A, Gosden T, Forland F, Kristiansen IS, Sergison M, et al. (2000) Target payments in primary care: effects on professional practice and health care outcomes. Cochrane Database Syst Rev Issue 3. Art. No.: CD000531: CD000531.
25. Peñaloza B, Pantoja T, Bastías G, Herrera C, Rada G (2011) Interventions to reduce emigration of health care professionals from low- and middle-income countries. Cochrane Database Syst Rev 9: Issue 9. Art. No.: CD007673.
26. Kongnyuy E, van den Broek N (2009) Audit for maternal and newborn health services in resource poor countries. BJOG 116: 7-10.
27. Jamvedt G, Young JM, Kristoffersen DT, O'Brien MA, Oxman AD (2006) Audit and feedback: effects on professional practice and health care outcomes. Cochrane Database of Systematic Reviews Issue 2. CD000259.pub2.
28. Ovretveit J (1992) Health service quality: an introduction to quality methods for health services: Blackwell Special Projects.
29. Maxwell RJ (1992) Dimensions of quality revisited: from thought to action. Quality in health care 1: 171.
30. Committee on Quality of Health Care in America IoM (2001) Crossing the quality chasm: A new health system for the 21st century: National Academies Press.
31. Donobedian A (1980) Explorations in Quality Assessment and Monitoring, The Definition of Quality and Approaches to its Assessment, Mick. Health Administration Press, Ann Arbor.
32. Raven J, Hofman J, Adegoke A, van den Broek N (2011) Methodology and tools for quality improvement in maternal and newborn health care. International journal of gynaecology and obstetrics 114: 4-9.
33. Bruce J (1990) Fundamental elements of the quality of care: a simple framework. Stud Fam Plann 21: 61-91.
34. Hulton LA, Matthews Z, Stones RW (2007) Applying a framework for assessing the quality of maternal health services in urban India. Soc Sci Med 64: 2083-2095.
35. Souza JP, Gulmezoglu AM, Vogel J, Carroli G, Lumbiganon P, et al. (2013) Moving beyond essential interventions for reduction of maternal mortality (the WHO Multicountry

Survey on Maternal and Newborn Health): a cross-sectional study. Lancet 381: 1747-1755.

36. UNFPA (2012) Trends in maternal mortality: 1990 to 2010. http://www.unfpa.org/webdav/site/global/shared/documents/publications/2012/Trends_in_maternal_mortality_A4-1.pdf.
37. Lim SS, Dandona L, Hoisington JA, James SL, Hogan MC, et al. (2010) India's Janani Suraksha Yojana, a conditional cash transfer programme to increase births in health facilities: an impact evaluation. Lancet 375: 2009-2023.
38. International. CSAEal (2013) Ethiopia Demographic and Health Survey 2011. Addis Ababa, Ethiopia and Calverton, Maryland, USA: Central Statistical Agency and ICF International.
39. Shea BJ, Grimshaw JM, Wells GA, Boers M, Andersson N, et al. (2007) Development of AMSTAR: a measurement tool to assess the methodological quality of systematic reviews. BMC Med Res Methodol 7: 10.
40. World Health O (2012) WHO: Trends in maternal mortality: 1990 to 2010: WHO, UNICEF, UNFPA and The World Bank estimates. http://wwwunfpaorg/webdav/site/global/shared/documents/publications/2012/Trends_in_maternal_mortality_A4-1pdf.

Figure Legend:

Figure 1 Map with countries by category according to their maternal mortality ratio (MMR, death per 100 000 live births), 2010

Figure 2 Quality of care model

Figure 3 Conceptual Framework

Figure 1 Map with countries by category according to their maternal mortality ratio (MMR, death per 100 000 live births), 2010

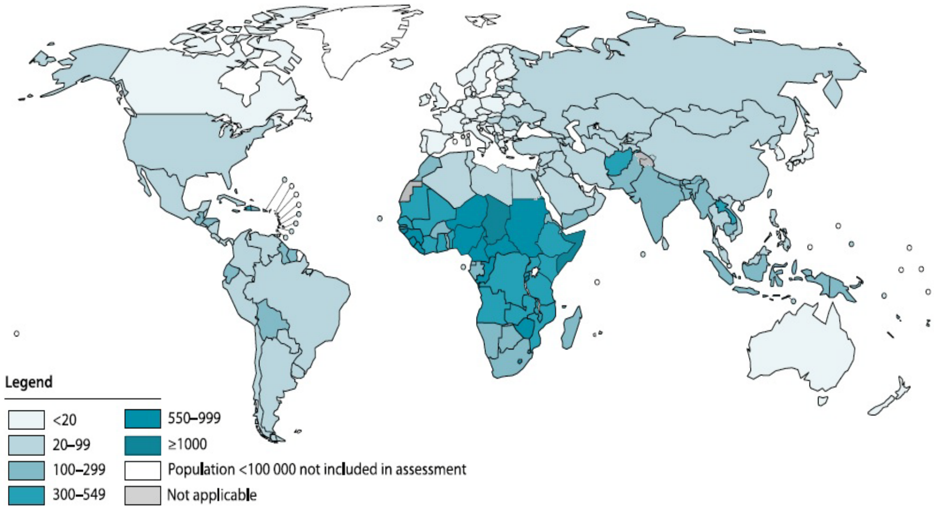

Source: Trends in maternal mortality:1990 to 2010 by WHO, UNICEF, UNFPA and The World Bank estimates[40]

Figure 2Quality of care model

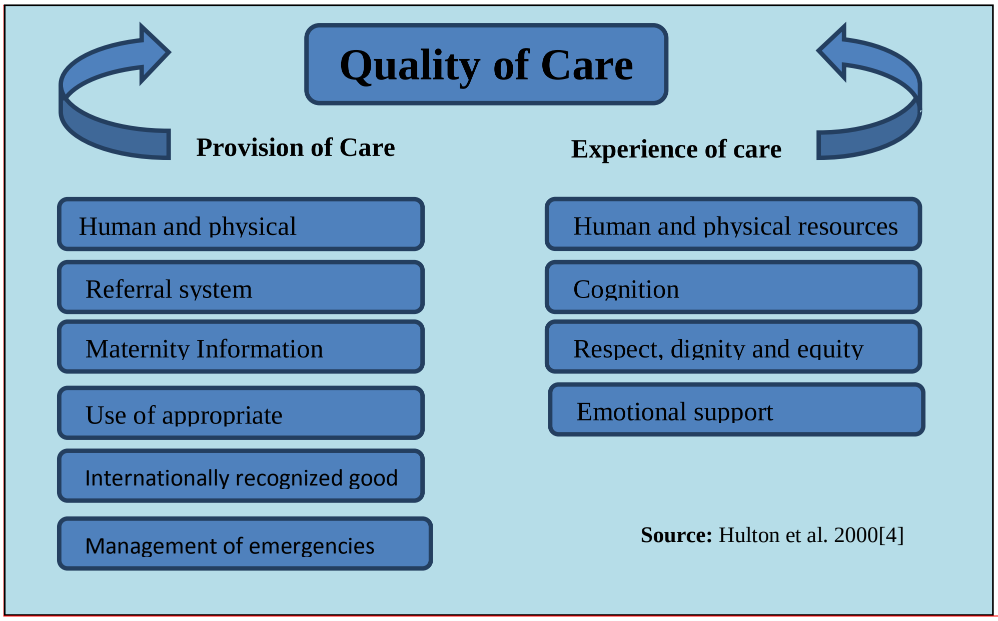

Comment [FA4]: PLEASE CLARIFY/CORRECT "INTERNATIONALL RECOGNIZED GOOD"

Prof Jos van Roosmalen

The authors have to address the following issues:

1. The distinction in district, community and facility level inputs is important, but strange enough on page 10 and also in figure 3 ”accountability” is not mentioned at the facility level.
   One of the interventions which address accountability at the facility level is audit and feedback, which is not found in the conceptual framework (figure 3) nor in the quality of care model (figure 2).

Thank you for this suggestion. We have included governance and accountability at the district, as well as audit and feedback facility level in the conceptual framework (FIG 2 in the revised paper).

Unfortunately, we could not modify figure 2 in the former version, because it is not a model we developed, but we have removed it from the manuscript and only describe it in the text.

2. This seems to be a systematic review of systematic reviews, which I think is a methodological weakness. First because it will miss papers which are not taken up by previous reviews and second because it will make this review already outdated at the time of publication, because it does not include recent relevant papers which could not be included in the old reviews, but should have been included in this review. See below some of the relevant papers which are not included; and there will be more relevant papers, because I have not done a search, but just happen to know these few ones.

Your assessment of what we did and the limitations you highlight are correct. Doing a systematic review of systematic reviews offers, however, important advantages: 1) it builds on the conclusions of rigorous reviews of multiple quality intervention studies in different settings; 2) it avoids duplication of work done by other researchers; 3) it allows for a much faster review.

We made a special effort to avoid missing important, more recent papers. Inevitably, we needed to limit the review to papers published until a certain date, and those published later than that could not be included. This would happen with any review. We will include an analysis of the limitations of this approach in paper 5.

3. When the quality of care is referenced on page 4 (13, 14, 15) I miss recent references of papers which address the use of audit and substandard care at facility level in districts in rural Africa instead of coming from tertiary care hospitals. To give just a few examples I know from my own involvement: Van den Akker T, et al. Reduction of severe acute maternal morbidity and maternal mortality in Thyolo District, Malawi: the impact of obstetric audit. PLoS One 2011; 6 (6): e20776; Nyamtema AS, et al. Using audit to enhance quality of maternity care in resource limited countries: lessons from rural Tanzania. BMC Pregnancy Childbirth 2011 Nov 16; 11: 94.

Please see comment on review of reviews and timing of publications above. You might not be able to find individual studies in the paper since we have conducted and overview of the systematic reviews

4. Last sentence of first par. of introduction (pag. 3) reads a bit strange: women who avoid maternal death as if they can actively avoid that, please correct; the authors rightly ask attention for the many women with morbidities and then continue to state: “over 80% of these deaths …”, while they do not address deaths in this sentence. This could easily be corrected.

Following your recommendation, the paragraph now reads: “Although there have been substantial declines in the annual number of maternal deaths since 1990, an estimated 273,500 women die every year as a result of maternal causes.[1] Sub-Saharan Africa (56%) and Southern Asia (29%) account for 85% of the global burden[2], while at the country level, two countries contribute a third of global maternal deaths: India at 19% (56 000) and Nigeria at 14% (40 000) (**Figure 1**). Among women who survive childbirth, approximately 10 million will suffer from complications related to pregnancy and childbirth [3,4]. Many of these conditions or deaths could be prevented through timely interventions that have proven to be effective and affordable [5-7].”

5. On page 5, the last sentence of the first par.: “Targeted and collaboratively designed approaches to intervention delivery need to be highlighted ...” People who work at the grass root level and in the first referral level facilities really do not understand what the authors want to say here. One of the problems is: do they address the delivery of interventions, while delivery in the facilities address the actual process of birth!

Thank you for pointing this out. This section has been completely re-written to clarify what we meant to say.

6. On page 7 under “Why facilities, why this frame?” the authors address the issue of “dystocia” as related to “the third trimester of pregnancy”. I cannot follow this because different from e.g. placenta previa and preeclampsia, dystocia cannot be predicted and will only be detected during a proper trial of labour.

Thank you for identifying this mistake. We changed this paragraph. Please see new version of first paragraph of page 10.

7. On page 9 in panel 1 under Human Resources-Training under Community Level inputs I miss training on the spot, let us say in the labour wards instead of conferences, workshops, lectures, seminars, symposia and courses. Such training can be considered as a “disease”, mainly being pursued for financial remunerations, while training at the spot is at the same time “service delivery”. Strange is also that this type of training is not addressed at the facility level.

Thank you for your comment. Under human resources training, we have now included on-the-job training. We have also added training methodologies at the facility level for well performing and motivated workforce.

8. I find attention for influenza vaccination (panel 1, page 9) and for the use of mammogram (page 10) unbalanced and mainly indicating high resource countries bias. And if we want to address such issues why not mentioning hepatitis B-vaccination and VIA for cervical carcinoma?

We agree that currently these interventions are practiced in high income countries. However, our aim was not to recommend any particular intervention but rather focus on how to improve the uptake, coverage and quality of the interventions delivered. We consider your comment and have now added in the specific sections in paper 4 that these interventions are currently generalizable to high income settings.

9. In the types of outcomes (page 12) I miss the results of audit and the perception of health care worker's of such interventions.

We have now added providers' adoption of evidence-based interventions and compliance with desired practice and other provider-related aspects of quality of care under the outcomes

Responses to review by Shea

Abstract: The question/objective is not articulated.

We have re-written the abstract; thank you for this observation.

Introduction: The first paragraph contains information that highlights the problem. I suggest inserting a topic/introductory sentence that includes maternal and neonatal mortality. "Among women who avoid maternal death …" – can be revised.

We revised this paragraph and highlighted maternal and newborn mortality.

"and the trend is expected to continue as the under five deaths reduce disproportionately"(p.3) – what does this mean?

This statement was deleted.

The definition of "quality in health care" does not adequately feature current debates and contentions about quality of care.

We think we provide enough information about the selection of the definition and conceptual framework of quality of care we used for this review.

The content of the 1st paragraph on page 5 is good.
The data in the ***Introductory*** section are not presented systematically or well, and the flow between paragraphs is not effective, which hampers the overall coherence of the paper.

We have rewritten the introduction to make it flow better.

Conceptual Framework: The Conceptual Framework presented on page 20 appears to follow an abridged Logic model and encompasses salient aspects of quality care. It would be worth considering streamlining points iv and v in the *District Level Inputs* component. One example would be Health Information Systems rather than *Service-Infrastructure-Electronic health records/electronic communication*.

We made these changes, thank you.

The framework will benefit from strategic revision that takes into account current terminology, like the Health Workforce and Community Engagement, for example.

Thank you for these suggestions. We have replaced "human resources" with "health workforce" and "community mobilization" with "community engagement".

Regarding quality health care, it is not only the training component or task-shifting that is key; it is unclear why these two features are emphasized.

HR training is imperative for clinically appropriate care. Task shifting is considered an effective way to expand clinical services in low resource settings. We offered these two approaches as examples, among many others that we could have provided.

Panel 1: Definition of quality care components:-
The Leadership and Supervision definition does not adequately incorporate a leadership definition.

In Panel 1, we define leadership as the provision of monitoring, guidance and feedback on matters of personal, professional and educational development in the context of the patient care.

Service Infrastructure-Information System – does this relate only to patient-related information?

In Panel 1 we define **Service Infrastructure-Information System as** electronic health records, i.e., existence of and access to electronically retrievable health records at the point of healthcare delivery. It may also include the related training components. Electronic communication included computerized communication, telephone follow-up and counseling, interactive telephone systems, after-hours telephone access, and telephone screening

Search Strategy: The search strategy for systematic reviews is explained in detail. It appears that the authors chose approaches that have an impact on frontline workers' implementation of interventions, and included search terms or strings embedded in the conceptual framework.
It is important to note that Cochrane Systematic Reviews include only Randomised Controlled Trials (RCTs) and do not include a wide variety of other research designs, like mixed method studies. General information about the study designs included in reviews other than Cochrane Systematic Reviews is recommended.

Agreed. Information regarding the study designs included in reviews is mentioned in the characteristics of included reviews.

The manuscript suffers from a lack of attention to scholarly writing. There are several language edits required throughout the paper, starting with the Abstract.
The manuscript needs complete language/editing revision.

We have completely revised the manuscript for language, editing and flow.

Responses by review by Sundby

Here is some feedback on the five papers, I met Viva yesterday and we discussed it.

In general, there are good things about the papers as they try to review which types of interventions have been proven (evidence wise) to make a difference in MCH care - at three different levels: community, hospital etc). That is actually what the papers are about. But as they are presented as a broader approach, and that's where we miss out. Papers like this have to be presented in a stronger theoretical framework, and the first and the final paper have to be totally re-written to obtain that task.

Paper 1 was extensively edited. To better explain the theoretical framework, we made several additions that discuss the three components (structure, process, and outcome) of the logic model.

Quality of care is complex. We have to differentiate between quality assessment, quality assurance and quality improvement. This has not been clarified in the papers.

Our framework and papers focus on approaches to quality improvement, not quality assurance. Quality assurance, requiring monitoring and evaluation, is key in quality improvement cycles- and are included in our framework, but are not our main focus.

Also, there is a vast amount of literature on methods both for quality assessment (Donabedian framework and others) and for quality improvement (quality improvement cycles). These are not at all referred to in the papers. Input, process, output, outcome quality aspects, as well as "whose perspective" has to be addressed, and also methodological challenges in assessing expectancy.

The paper was extensively revised to address these important comments.

Quality of Care is a normative concept. Therefore, one also has to address the issue of standards. Which standards of care are we pointing to? Whose policies, norms and regulations do we relate to? "Gold standards" and interim standards. To measure quality of care is always to measure "what is" against "what should be" or "what could ideally be".

The first and last papers do not take up such issues.

I suggest either to reformulate the intention of the papers or to expand them somewhat to give a scientific framework around the whole concept of quality of care, and also to mention some known common shortcomings in MCH in low resource countries.

We adopted the IOM definition of quality care. In theory, achieving all of these process

indicators would result in improved maternal/newborn outcomes.

Quality improvement is an iterative process, and understanding the causal pathways that impact processes and outcomes is fundamental for change. The intention of the framework is to provide a flexible, intuitive tool, which policy makers, program implementers, providers and communities can adapt to their particular context. As the inputs at the district, community and facility are interconnected, this would allow, for example, a provider to examine the district and community inputs that are impacting his/her ability to provide quality care. The framework, in and of itself, has been left flexible, and we encourage adaptations to fit the realities on the ground in low resource settings.

The intention of this framework is to provide a conceptual overview of connections, synergies and relationships between different system levels of input on processes and outcomes. The series of papers that follow carefully examine the evidence base of interventions at each of these three input levels that impact the provision of quality maternal/newborn health services.

The known shortcomings in MCH in low resource settings are many, must be understood from multiple input levels.

Responses to Althabe review

I very much liked the series and this introductory paper. Analyzing and summarizing the existing evidence on interventions to improve Q of C is a high priority for global health. The conceptual framework is appropriate, very comprehensive, with a good literature review. Methods are strong and well described. I only have very minor issues to comment:

- Order of the inputs description: District, community, and facility inputs are described in different order in paper 1 (ie. Panel 1 and fig 3), and compared to paper 5.

This has been fixed, thank you!

- At pag 7, second paragraph, 3rd sentence, I suggest a minor change: *Although the latter is the most hazardous to women, the third trimester of pregnancy is also a the period of higher occurrence of pregnancy complications, including placenta previa and other causes of hemorrhage, pre-eclampsia and eclampsia, and dystocia, among other conditions.*

We fixed this sentence and added labor as a high risk period. Thank you.

- Search strategy: please include time period of literature search.

Added

- Please check Fig 2: “internationally recognized good” not clear what it means

Hulton’s framework draws almost exclusively from the RCOG guidelines that include 8 main criteria. They also acknowledge that there are other sources from which good-practice criteria can be identfied, with the WHO as an example. These are outlined on table 5, page 57 of the linked document:

http://eprints.soton.ac.uk/40965/1/12757_Matthews.pdf

The framework we used was taken from the paper.

- Page 13 says "*The next part of this 5 series review will focus on interventions and findings of District Level Inputs on QoC for women and newborns* However the series order seem to include Community interventions as the second paper. Please revise.

We have ordered the community, district, and facility level inputs so that they follow the flow of the series. We will ensure paper 5 flows correctly, too. Thank you for pointing this inconsistency out.
